# Inherent Antibacterial Properties of Biodegradable FeMnC(Cu) Alloys for Implant Application

**DOI:** 10.1021/acsabm.3c00835

**Published:** 2024-01-22

**Authors:** Birgit Paul, Annika Kiel, Martin Otto, Thomas Gemming, Volker Hoffmann, Lars Giebeler, Bernhard Kaltschmidt, Andreas Hütten, Annett Gebert, Barbara Kaltschmidt, Christian Kaltschmidt, Julia Hufenbach

**Affiliations:** † Leibniz Institute for Solid State and Materials Research Dresden, Helmholtzstr. 20, 01069 Dresden, Germany; ‡ Department of Cell Biology, Faculty of Biology, Universität Bielefeld, Universitätsstraße 25, 33615 Bielefeld, Germany; § Institute of Materials Science, Technische Universität Bergakademie Freiberg, Gustav-Zeuner-Str. 5, 09599 Freiberg, Germany; ∥ Department of Thin Films and Physics of Nanostructures, Center of Spinelectronic Materials and Devices, Faculty of Physics, Universität Bielefeld, Universitätsstraße 25, 33615 Bielefeld, Germany

**Keywords:** bioresorbable metal, implant-related infections, bactericidal effect, GD-OES, TEM

## Abstract

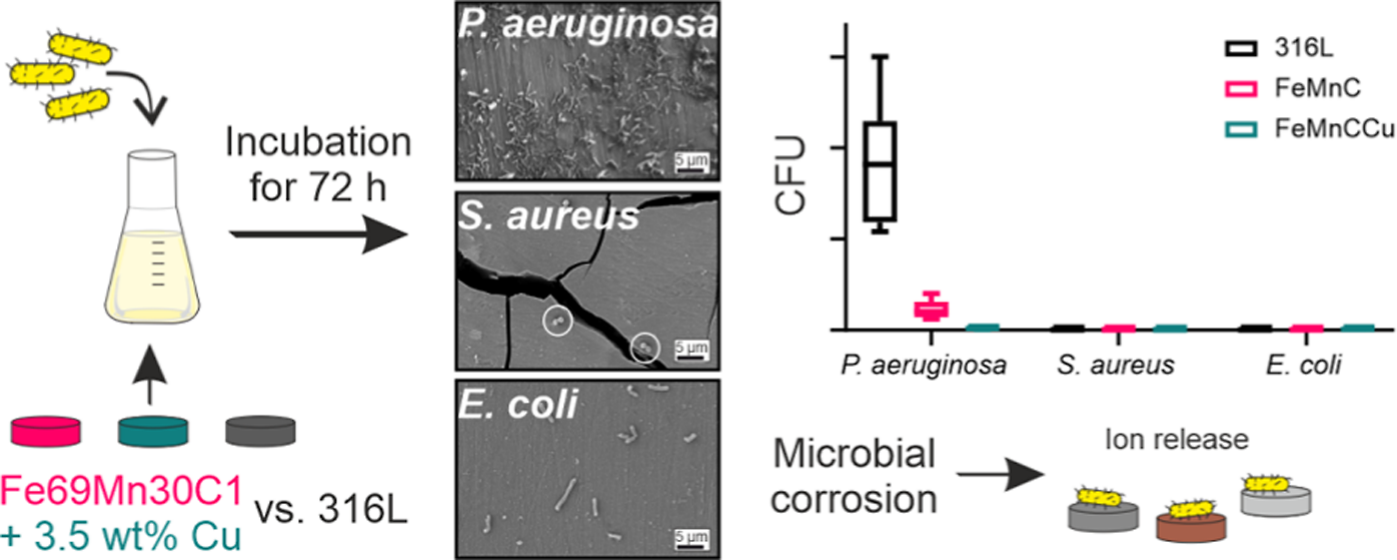

Implant-related infections or inflammation are one of the main reasons for implant failure. Therefore, different concepts for prevention are needed, which strongly promote the development and validation of improved material designs. Besides modifying the implant surface by, for example, antibacterial coatings (also implying drugs) for deterring or eliminating harmful bacteria, it is a highly promising strategy to prevent such implant infections by antibacterial substrate materials. In this work, the inherent antibacterial behavior of the as-cast biodegradable Fe69Mn30C1 (FeMnC) alloy against Gram-negative *Pseudomonas aeruginosa* and *Escherichia coli* as well as Gram-positive *Staphylococcus aureus* is presented for the first time in comparison to the clinically applied, corrosion-resistant AISI 316L stainless steel. In the second step, 3.5 wt % Cu was added to the FeMnC reference alloy, and the microbial corrosion as well as the proliferation of the investigated bacterial strains is further strongly influenced. This leads for instance to enhanced antibacterial activity of the Cu-modified FeMnC-based alloy against the very aggressive, wild-type bacteria *P. aeruginosa*. For clarification of the bacterial test results, additional analyses were applied regarding the microstructure and elemental distribution as well as the initial corrosion behavior of the alloys. This was electrochemically investigated by a potentiodynamic polarization test. The initial degraded surface after immersion were analyzed by glow discharge optical emission spectrometry and transmission electron microscopy combined with energy-dispersive X-ray analysis, revealing an increase of degradation due to Cu alloying. Due to their antibacterial behavior, both investigated FeMnC-based alloys in this study are attractive as a temporary implant material.

## 1 Introduction

Implant-related infections are of increasing concern because they can lead to local tissue damage, systemic spread of pathogens, and implant failure.^1^ While many patients even get their implants in an advanced age, the number of applied implants increases as well as the infectious risk. This risk of implant-related infections varies on the clinical issue whereby cardiovascular implants imply the highest mortality in the US in 2001, e.g., vascular grafts have an average infection rate of 4% and ventricular assist devices even as high as 40%.^2^ In orthopedics, implant-related infection is a widely occurring and the most serious complication.^3^ Implant-related infections are extremely difficult to treat as a well-established antibiotic therapy is needed for a longer duration and repeated surgical procedures may have to be conducted.^2^ Bacteria can directly enter the implantation site during the surgical procedure or through circulation in blood from another infection site and open wounds.^1^ However, due to the excessive use of antibiotics, multidrug-resistant microorganisms are increasingly prevalent, threatening public health.^4^ An estimated 10 million deaths will be caused by multidrug-resistant infections by 2050 if no action will be applied to decelerate drug resistance.^5^ The European Union is working on “Research and Innovation Objectives of the European Partnership on One Health Antimicrobial Resistance” (EUP OH AMR) to support new strategies to overcome this challenge, which is aimed to be started presumably at the beginning of 2025.

Biofilm formation is described as follows:^3,6−8^ the initial step in implant-related infection is the reversible adhesion of bacteria onto the surface within the first 1–2 h after implantation. This process is followed by surface coverage and expression of the extracellular matrix, leading to irreversible bonding of the bacteria to the implant. Subsequently, bacteria colonize into microcolonies, eventually leading to a biofilm after about 24 h. As things develop, columnar and tower structures will grow, followed by dissemination of the biofilm and dispersion for colonization of new surfaces. *Staphylococcus aureus* is one of the most common Gram-positive bacteria in orthopedic implant-related infections, whereby infections with aerobic Gram-negative bacteria like *Pseudomonas aeruginosa* or *Escherichia coli* steadily increase.^3,7^ Furthermore, *S. aureus* and *P. aeruginosa* are the main two bacteria causing coronary stent infections within the first 10 days after stent implantation, which is generally treated by antibiotics.^9^ However, later infections caused by methicillin-resistant *S. aureus* might also need surgical treatment.^9^ On the other side, *E. coli* is the most prominent pathogen to cause urinary tract infections.^10^

One way to reduce local implant-related infections is to develop implants with antimicrobial properties.^3^ The most common strategies to inhibit bacteria adhesion, to eliminate bacteria or to prevent biofilm formation on implants are coatings (e.g., antifouling polymers, antimicrobial peptides, and biodegradable drug-loaded polymers or inorganics), surface treatments (e.g., ion implantation, sandblasting, etching, and nanostructuring to generate, e.g., nanotubes, nanoporosity, and nanopillars), or alloy design.^3,8,11−16^ Alloys with inherent antibacterial properties have several advantages over coatings or surface treatments, such as longer, stable and broad-spectrum antibacterial effect, inexpensive manufacturing by conventional processing methods including easier sterilization treatment, and no known drug resistance.^8^ There are a few elements that are known for their antimicrobial activity. The most common cations with antibacterial effects are Ag, Cu, or Zn.^3,8^

The antibacterial effect of iron has been well-known and studied for a long time in, e.g., natural ferruginous clays.^17^ One underlying molecular mechanism might be the formation of reactive oxygen species (ROS) during the corrosion reactions of Fe. Corrosion can lead to the formation of divalent Fe ions and H_2_O_2_. Both components react according to the Fenton reaction and can generate hydroxyl radicals.^18^ The formation of such hydroxyl radicals was verified for pure Fe and Fe-22Mn-0.6C immersed for 30 min in phosphate-buffered solution.^18^ Hydroxyl radicals are toxic for cells due to lipid peroxidation, e.g., oxidation of the cell membrane components, which destroys the cell membrane. Furthermore, intracellular uptake of soluble Fe(II) ions is induced, which could increase the ROS concentration inside the bacterial cells.^17^ Subsequently, this reaction promotes the oxidation of macromolecules and finally the cell death.^17^

Cu is even more reactive than Fe in producing ROS.^19^ Besides ROS generation, Cu ions can inhibit bacteria by cell membrane damage or direct contact killing.^3^ However, the exact effect of Cu in killing bacteria still awaits complete understanding. For stainless steel alloyed with Cu, mainly two contributions are stated: either the interaction with released Cu ions or direct contact with Cu, which can induce bacterial cell death. A few possible mechanisms are discussed in literature:^20,21^ (i) metabolization of Cu ions, thus forming toxic metabolites; (ii) production of ROS in auto-oxidation or Fenton reactions catalyzed by Cu, leading to the penetration and disruption of cell membranes; (iii) entry of Cu ions into the cell, e.g., via ion channels in the cell membrane, that could promote ROS formation in the cytoplasm and subsequent damage of the cell membrane from inside; (iv) interaction of Cu ions with thiol-containing proteins leading to their deactivation and bioactivity loss; (v) morphological changes involving detachment of the cytoplasmic membrane from bacterial cell wall; (vi) different distributions of charges near the membranes can result in cell membrane damage; and (vii) positively charged Cu ions may interfere with the negatively charged DNA and inhibit bacterial multiplication. The introduction of Cu to various implant materials for permanent load-bearing application, e.g., stainless steels,^22^ titanium alloys,^23^ or Co-based alloys^24^ improved their antibacterial effect. In addition, it could be shown that Cu may also stimulate blood vessel growth and new bone formation.^25^

Therefore, Cu also shows a high potential as an alloying element for degradable systems. However, as those materials should release Cu ions as they degrade, lower Cu contents would be sufficient. Besides Mg-based^26,27^ and Zn-based,^28^ Fe-based alloys^29−32^ are also widely investigated for the use as temporary implant material with high potential for future clinical application. Regarding degradable Fe-based implant materials, only a few studies investigate the alloying with Cu. The majority of those studies focus on powder metallurgy or mixed powders for additive manufacturing of Fe with up to 10.1 wt % Cu^33,34^ or Fe–Mn with up to 10 wt % Cu.^35,36^ Extracts of various Fe–Cu as well as Fe–Mn–Cu alloys were shown to have a bactericidal effect on *E. coli* after 24 h of incubation.^33−36^ Only the group of Yang et al.^37,38^ investigated cast Fe-based alloys, Fe-30Mn-1C alloys with up to 1.5 wt % Cu. The as-cast FeMnC-based alloy with 0.8 wt % Cu effectively reduced *S. aureus* after 24 h incubation and reached a bactericidal rate of 99%.^37^ These FeMn-based alloys are more favorable than Fe-based alloys due to their good processability combined with enhanced strength and a higher *in vitro* degradation rate. They were tested as potential material for bone, cardiovascular, and urinary applications.^30,37,39,40^

*In vitro* corrosion mechanisms are quite well understood for FeMn-based alloys in chloride-ion containing physiological inorganic salt solutions, such as SBF or Hank’s balanced salt solution (HBSS).^41,42^ Shortly, the initial anodic corrosion reactions are the oxidations of Fe and Mn to ions, which are partially released in the electrolyte. The simultaneous cathodic reaction reduces the level of dissolved O in water by consuming the electrons to hydroxide ions and controls the metal oxidation. Subsequently, both cations and anions react to form insoluble metal oxides or hydroxides. With some distance to the bulk metal surface, metal ions (for convenience, only Fe will be considered subsequently, but it also applies for Mn) are further oxidized from Fe^2+^ to Fe^3+^ to form hydrous ferric oxides or hydroxides resulting in different stacks of the degradation layer from the bulk metal interface to the outer degradation layer: FeO·*n*H_2_O, Fe_3_O_4_·*n*H_2_O, and Fe_2_O_3_·*n*H_2_O. Cu is initially oxidized into Cu^+^ and forms CuCl in chloride-containing solutions. With proceeding corrosion reactions various chloro-complexes as well as oxides and phosphate-containing complexes are generated depending on local pH value variations and local Cu ion concentration.^43^

As the degradation proceeds, Ca, Fe, and Mn phosphates and carbonates precipitate on the surface. These reactions may be caused by a local increase of the pH due to the generation of hydroxide ions in the cathodic reaction.^44^ The precipitation of such compounds leads to the formation of a complex and relatively dense degradation layer.^42^ When the layer is able to grow to a certain extent, it may act passivating which reduces ion diffusion properties and therefore the degradation rate due to mass transport limitations of the reactive species.^42^

In this study, the influence of 3.5 wt % Cu addition on Fe69Mn30C1 regarding the microstructure, degradation behavior, and antibacterial effect is investigated in comparison to clinically applied 316L stainless steel for the first time. The influence of alloy composition on the microstructure, corrosion mechanisms, and related antibacterial behavior was investigated. Short-term corrosion activity of both FeMnC-based alloys was electrochemically analyzed by potentiodynamic polarization (PDP). Furthermore, the initial formation of the degradation layers was studied by GD-OES as well as by TEM and EDX analysis. For the antibacterial behavior, three bacterial strains relevant for implant-related infections, *P. aeruginosa*, *S. aureus,* and *E. coli* were applied to analyze the influence of the alloy modification on different types of bacteria.

## 2 Materials and Methods

### 2.1 Alloy Preparation and Chemical Analysis

The examined alloys Fe69Mn30C1 (in wt %; FeMnC) and Fe65.5Mn30Cu3.5C1 (in wt %; FeMnCCu) were prepared by melting the pure elemental constituents (Fe: 99.98%, Mn: 99.75%, C: 99.5%, and Cu: 99.9%) under an argon atmosphere in an induction furnace (Balzers, Hanau, Germany). At a temperature of about 1500 °C, the melt of both alloys was poured into a Cu mold for the resulting plate-shaped ingot with dimensions of 15 × 70 × 120 mm^3^. A commercial austenitic corrosion-resistant AISI 316L stainless steel (Viraj Profiles Pvt. Ltd., Mumbai, India) was used as the reference.

The nominal and experimentally determined chemical compositions of the cast ingots are presented in Table 1. Carrier gas hot extraction (CGHE; G4 ICARUS, Series 2, Bruker Corporation, Billerica, MA, U.S.A.; *n* = 3) was conducted to analyze the carbon content, and inductively coupled plasma optical emission spectroscopy (ICP-OES; iCAP 6500 Duo View, Thermo Fisher Scientific Inc., Waltham, MA, U.S.A.) was used for analyzing the element concentrations Fe, Mn, and Cu (*n* = 3).

**Table 1:** Nominal and Experimentally Determined Chemical Compositions of the Investigated FeMnC(Cu) Alloys and of AISI 316L Reference

nominal/experimental composition	Fe/wt %	Mn/wt %	C/wt %	Cu/wt %	Cr/wt %	Ni/wt %	Mo/wt %	Si/wt %
AISI 316L	68.44 ± 0.43	1.62 ± 0.01	0.0211 ± 0.003	0.38 ± 0.0004	16.80 ± 0.09	10.19 ± 0.08	2.08 ± 0.02	0.39 ± 0.03
Fe69Mn30C1 (FeMnC)	70.66 ± 0.44	28.47 ± 0.21	0.966 ± 0.006					
Fe65.5Mn30Cu3.5C1 (FeMnCCu)	64.53 ± 0.32	30.49 ± 0.16	1.076 ± 0.010	3.55 ± 0.02				

### 2.2 Microstructural Characterization and Elemental Distribution

Embedded FeMnC(Cu) samples were ground to P4000 with SiC grit paper, polished up to 0.25 μm diamond suspension, and in the final step with MasterMet 2 (Buehler, Illinois Tool Works Inc., Lake Bluff, IN, U.S.A.). The microstructure was investigated by scanning electron microscopy (SEM; Leo 1530 Gemini, Carl Zeiss Microscopy Deutschland GmbH, Oberkochen, Germany) with backscatter electron imaging (BSE) and energy-dispersive X-ray analysis (EDX; Xflash4010, Bruker Corporation) for the detection of Fe, Mn, C, and Cu.

X-ray diffraction analysis was performed in the transmission mode (XRD; STOE Stadi P, STOE & CIE GmbH, Darmstadt, Germany; Ge(111) monochromator; Mo Kα1 radiation; Mythen 1K detector) on P4000-ground FeMnC(Cu) pieces with a thickness of approximately 80 μm in the range of 10° ≤ 2θ ≤ 60°. Before the measurements, the samples were fixed to X-ray transparent polyacetate films with designated glue consisting of 50 v/v amyl acetate (Acros Organics-Thermo Fisher Scientific Inc.) and collodion solution (Merck KGaA, Darmstadt, Germany). The measured data were analyzed with LeBail fit with the program Fullprof in the WinPlotR software platform.^45^ The basic structure data for the LeBail analysis was taken from the structure model for Fe with the space group *Fm*3̅*m* as published by Westgren and Lindh.^46^

Transmission electron microscopy (TEM; Tecnai F30, FEI Company-Thermo Fisher Scientific Inc.) was performed with 300 kV in the scanning mode using a windowless EDX detector (TEAM Octane T Optima, AMETEK Inc., Berwyn, PA, U.S.A.) for elemental analysis of samples after immersion for 24 h in tryptic soy broth (Millipore-Merck KGaA) solution with additional 1% glucose (d-(+)-glucose monohydrate; Sigma-Aldrich—Merck KGaA) (TSB) at 37 °C. The electrolyte TSB consists of 17.0 g/L casein peptone, 3.0 g/L soya peptones, 5.0 g/L sodium chloride, 2.5 g/L dipotassium hydrogen phosphate, and 3.5 g/L glucose. The specimens for TEM investigations were prepared as lift-out lamellae by a Ga ion beam using a focused ion beam microscope (FIB, Helios CX5, Thermo Fisher Scientific Inc.).

Glow discharge optical emission spectrometry (GD-OES; GDA750HR, Spectruma Analytik GmbH, Hof, Germany) was conducted on cylinders of 8 mm diameter and 6 mm height to get elemental depth profiles of samples after immersion for 24 h in TSB solution at 37 °C. Immersion time had to be reduced compared to bacterial tests (see Section 2.5), since Cu-containing samples could not be measured due to their porous degradation layer. The following discharge parameters were applied: 500 V anode voltage of the free running radiofrequency generator and 2.7 hPa Ar pressure using a modified universal sample unit to sputter craters with a 2.5 mm diameter at the surface. The following emission lines were recorded: Fe 371 nm, Mn 403 nm, Cu 327 nm, C 165 nm, Ca 393 nm, S 180 nm, N 149 nm, O 130 nm, and H 121 nm. After the measurements, the depth of the sputter crater was measured with a profilometer, and an erosion rate of about 4 μm/min was calculated as a rough estimation.

### 2.3 Electrochemical Studies

For each alloy, cylindrical samples of 8 mm diameter and height of 6 mm were embedded in epoxy resin forms. The specimens were ground to P2500 with a SiC grit paper, cleaned in an ultrasonic bath in pure ethanol for 10 min to remove the debris, and then placed in the cell setup. Samples were reused up to three times after repeated grinding and cleaning.

For determining the short-term corrosion properties, the electrochemical measurements were carried out in TSB solution with a three-electrode cell in a rotating disc setup connected to a rotator (A636A, AMETEK Inc.) and a potentiostat (SP-300, BioLogic, Seyssinet-Pariset, France). The nominal exposed area of the working electrodes (316L or FeMnC(Cu)) was 0.5 cm^2^. A platinum sheet served as the counter electrode, whereas for the reference electrode a saturated calomel electrode [SCE = 0.241 vs SHE (20 °C)] was placed in a Luggin capillary close to the working electrode to minimize the ohmic resistance contribution of the electrolyte. The rotation speed of the rotating disk electrode (RDE) was set at 500 rpm. First, the testing protocol consisted of stabilizing the open circuit potential (OCP) for 3 h followed by a PDP measurement starting from the cathodic site at −60 mV versus OCP and ending at +250 mV versus OCP with a scan rate of 1 mV/s. The tests were performed in triplicate at 37 ± 1 °C under atmospheric conditions. The pH value was determined before and after each measurement using a pH meter with a glass membrane electrode (VWR International GmbH, Darmstadt, Germany).

The evaluation of the corrosion rate is hard to quantify for FeMnC-based alloys as no well-defined Tafel regions are visible over at least one decade of current density on their anodic branch slope and therefore Tafel extrapolation should not be performed.^47^

### 2.4 Immersion Tests

Static immersion tests were performed for 72 h at 37 °C to analyze the initial corrosion steps in tryptic soy broth solution with an additional 1% glucose (TSB) solution. Samples (5 mm in diameter, 1 mm in height) were ground to P4000 SiC grit paper, ultrasonically cleaned with absolute ethanol, and subsequently sterilized with γ-irradiation. Fe, Mn, and Cu ion releases of the sterile-filtered (0.2 μm filter) solutions were evaluated by ICP-OES as described in more detail in a previous study.^41^

### 2.5 Bacterial Tests

Three bacterial species were selected for the biological assays. The strong biofilm producer *P. aeruginosa*, isolated from a domestic washing machine,^6^*S. aureus* (DSMZ 24167), and *E. coli* XL1-blue strain (Stratagene/Agilent, San Diego, CA, U.S.A.). The three bacterial strains were inoculated from frozen stock kept under −80 °C and cultured on fresh LB agar plates for 24 h prior to experimental use. From these plates, a single colony was picked and precultured in 10 mL of TSB medium (here without additional glucose) overnight.

To test the antibacterial activity of the samples in terms of viable colonies, a colony-forming unit (CFU) assay was performed as a direct quantification method. For each bacterium, five samples of each alloy were placed in a 100 mL Erlenmeyer flask inoculated with 80 mL of TSB, adjusted at an optical density of 0.1 at a wavelength of 600 nm, and incubated at 37 °C for 72 h. A sterility control for each alloy, containing only sterile TSB medium, was additionally performed. After the incubation period, planktonic cells were washed away by carefully submerging the samples three times in physiological saline (0.9% NaCl). The remaining biofilm was detached from the surface by vigorous vortexing for at least 1 min (Vortex Genie, Fisher Scientific GmbH, Schwerte, Germany) in 10 mL of physiological saline (0.9% NaCl). A serial dilution was performed, and 100 μL of each dilution was plated onto fresh TSB agar plates. The plates were incubated overnight at 37 °C. After incubation, colonies were counted, and the colony-forming units per mL were calculated. The bacterial suspensions for alloy incubation were sterile-filtered and the ion release was measured, as described above in Section 2.4.

For structural observation of biofilms with SEM, one sample of each alloy for all three bacterial strains was carefully washed in 0.9% NaCl solution, subsequently with distilled water, and was fixed in half-strength Karnovsky’s solution (2% paraformaldehyde, 2.5% glutaraldehyde) for 30 min. The fixed samples were dehydrated using 50, 70, 80, 90, 95, and 100% (v/v) grade ethanol followed by *t*-butanol. To improve the conductivity of the samples, they were sputter-coated with a layer of 4 nm ruthenium.

The size of the *E. coli* bacteria was measured by using the open source FIJI (ImageJ, National Institutes of Health, Bethesda, MD, U.S.A.) software. At least 10 SEM images per alloy were evaluated, and a total of 60 bacterial cells were measured in each case.

### 2.6 Statistical Analysis

The released Fe, Mn, and Cu ion concentrations are illustrated in boxplots showing the interquartile range with a line as the median and a circle as the median of three measured samples. For statistical analysis, a two-way analysis of variance was performed with posthoc Tukey test. The measured living bacteria (CFU/mL) are also shown in boxplots (*n* differs for each bacteria); whiskers display min. to max. values, box displays the interquartile range with a line at the median. For a uniform sorting out of extreme outliers, only the data points that were 80% around the median were plotted. For statistical quantification, the nonparametric Kruskal–Wallis-Test was performed. Bacterial length values are plotted individually for each alloy (*n* = 60) with a mean value line.

## 3 Results

Biodegradable FeMnC, as potential temporary implant material, was alloyed with 3.5 wt % Cu to increase the antibacterial activity. Both alloys were analyzed to investigate the influence of Cu addition on the microstructure, electrochemical behavior, formation of degradation layer, and ion releases as well as its influence on growth of the different Gram-negative and Gram-positive bacteria strains. Thereby, wild-type *P. aeruginosa*, *S. aureus,* and *E. coli* were applied.

### 3.1 Influence of Cu on Microstructure of Biodegradable FeMnC

**Figure 1:**
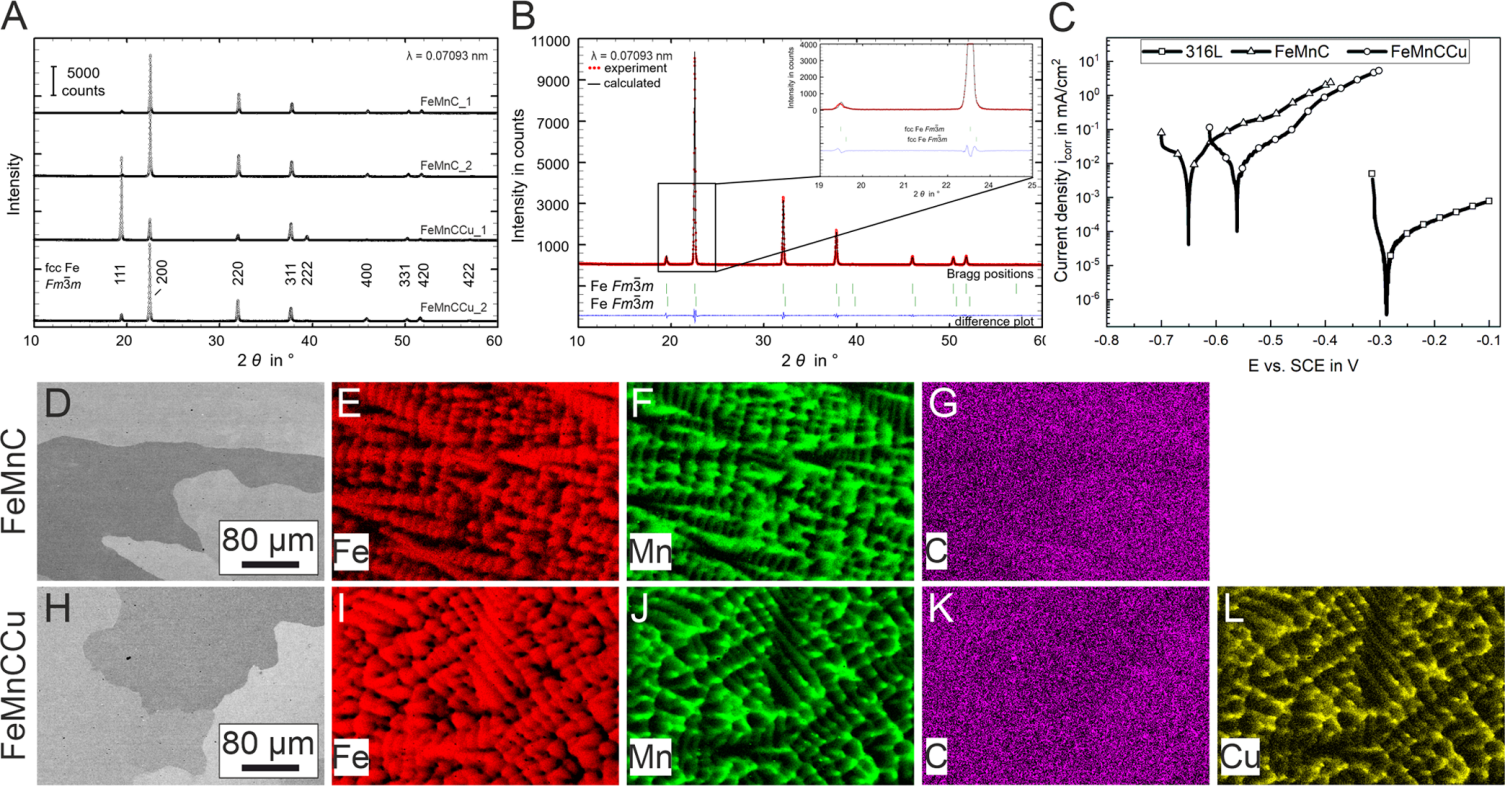
(A) XRD patterns of as-cast FeMnC and FeMnCCu, two samples in each case, with indexed austenitic phase. (B) XRD pattern of the sample FeMnC_1 after LeBail refinement: dotted red line shows the measured values and the solid black line shows the calculated values, identifying two fcc phases with different lattice parameters. (C) Representative potentiodynamic polarization (PDP) measurements of 316L, FeMnC, and FeMnCCu after 3 h of open circuit potential (OCP) stabilization in TSB electrolyte at pH 7.2 at 37 °C and under sample rotation of 500 rpm. (D) SEM image using backscattered electrons (BSE) of FeMnC and (E–G) corresponding EDX mappings of Fe, Mn, and C. (H) BSE-SEM image of FeMnCCu and (I–L) corresponding EDX mappings of Fe, Mn, C, and Cu.

In Figure 1A,B, the XRD patterns of both alloys for two samples, respectively, are exemplarily displayed. The analysis revealed a microstructure composed of face-centered cubic (fcc) austenite (space group *Fm*3̅*m*) in both alloys without amorphous parts or other phases within the methodical error. However, the LeBail analysis of the XRD spectra revealed two face-centered cubic phases with slightly different lattice parameters in sample FeMnC_1 (Table 2). The lattice parameter *a* of the second sample is between the lattice parameters of two austenitic phases in sample FeMnC_1. In FeMnCCu, the lattice parameter of the austenitic phase increases further.

**Table 2:** Lattice Parameters and Phase Contents of Identified Phases in Two Samples, Respectively, of As-Cast FeMnC and FeMnCCu Determined by the LeBail Fit of XRD Data

sample	space group	*a*/nm	*V*/nm^3^	phase content/wt %
FeMnC_1	*Fm*3̅*m*	0.363005(2)	0.047834(1)	95
		0.36075(3)	0.04695(1)	5
FeMnC_2	*Fm*3̅*m*	0.362420(4)	0.047999(2)	100
FeMnCCu_1	*Fm*3̅*m*	0.36413(2)	0.048278(7)	100
FeMnCCu_2	*Fm*3̅*m*	0.36407(1)	0.048258(5)	100

The as-cast state of both FeMnC samples and the FeMnCCu_2 sample indicates a textured microstructure since the relative peak intensity of 200 is larger than the one of 111. The latter one would be the reflex with the highest intensity for randomly oriented austenite crystals, which is the case for sample FeMnCCu_1. As the casting process is hard to control, this variety in segregation and texture can be found.

SEM images (Figure 1D–L) show a representative part of the as-cast alloys, visualizing grains in the cast state. The elemental distribution of Fe, Mn, C, and Cu was investigated by EDX analysis in the SEM. Within the grains a dendritic microstructure is visible. In Figure 1E–G, the EDX mappings of Fe, Mn, and C of FeMnC and in Figure 1I–L, the EDX mappings of Fe, Mn, C, and Cu of FeMnCCu are shown. In both alloys, Fe is enriched in dendritic regions and Mn in interdendritic regions. Carbon is relatively homogeneously distributed. Cu segregations were found in the interdendritic regions like Mn.

### 3.2 Effect of Cu Addition on Material-Bacteria-Interaction

#### 3.2.1 Initial Degradation Layer and Ion Release

Electrochemical analysis was applied for investigating the initial corrosion behavior in TSB at 37 °C under fluid flow condition as this is the standard method to evaluate the initial corrosion process dispassionately and quantitatively, e.g., the corrosion potential, in a relatively short analysis time. The PDP measurements of the corrosion-resistant 316L and the two biodegradable FeMnC-based alloys are shown in Figure 1C. After 3 h until OCP stabilization, the corrosion potential of the reference material 316L is with a value of −0.273 ± 0.018 V much more positive compared to that of both FeMnC-based alloys. This is mainly attributable to its spontaneously passivating nature, in contrast to the actively corroding FeMnC-based alloys. The addition of Cu shifted the corrosion potential to a more positive value of −0.568 ± 0.006 V compared to −0.651 ± 0.001 V for FeMnC. Compared to the corrosion current densities of the reference steel 316L the values for both biodegradable FeMnC-based alloys are about two orders of magnitude higher. This emphasizes their high initial degradation rate in the complex TSB electrolyte.

For both FeMn-based alloys, the anodic polarization regime is dominated by increasing current densities, revealing their active dissolution behavior. Within the error limits of the method, no Cu effect could be detected for the initial corrosion process. Furthermore, for both FeMnC-based alloys, the anodic curve branches, which represent the active dissolution, exhibit changes in their slope. In contrast to that, with increasing higher anodic potentials, the steep current density increases further for 316L, which indicates a passivity breakdown and the occurrence of pitting.

**Figure 2:**
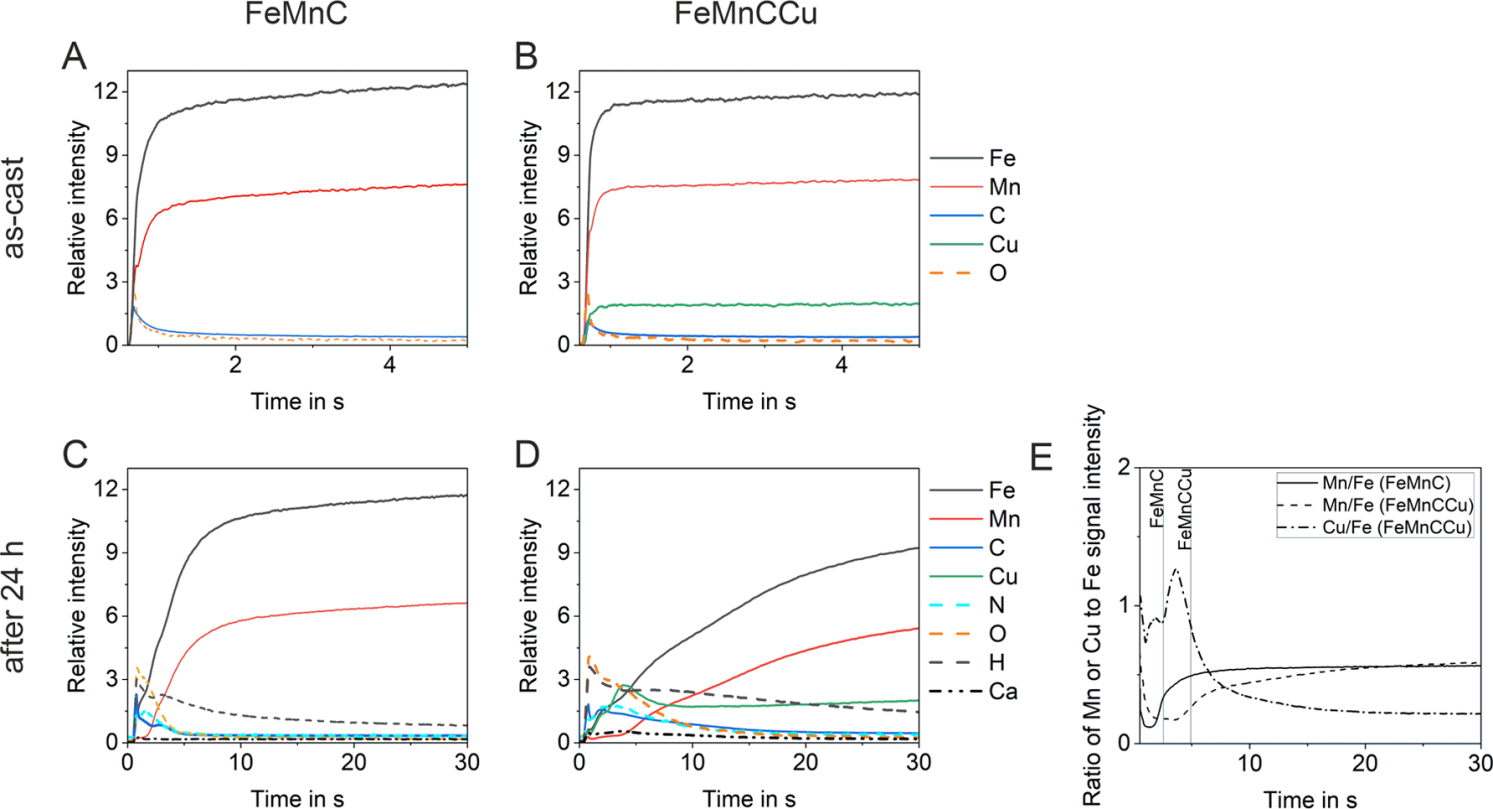
GD-OES depth profiles of (A,C) FeMnC and (B,D) FeMnCCu, (A,B) as-cast sample, and (C,D) after 24 h in TSB electrolyte. (E) Ratio of Mn or Cu signal intensity to the Fe signal intensity of both alloys after 24 h in TSB electrolyte. Vertical lines indicate the oxide layers of both alloys calculated by half of the O signal height.

For investigation of the forming initial degradation products, samples were analyzed after immersion in TSB for 24 h at 37 °C compared to the as-cast, ground state with GD-OES (Figure 2). As GD-OES is an indirect sputter procedure, the relative signal intensities in the diagrams cannot directly be correlated to the element concentration in the alloy.

However, both alloys have a thin native oxide layer as the O signal decreases rapidly within the first second (Figure 2A,B). For estimation of the oxide layer thickness, the half between the O signal peak and the saturation of the O signal^48^ as well as an erosion rate of 4 μm/min (see Section 2.2) were applied. For the native oxide layers, a thickness of about 10 nm for both alloys were estimated. As the estimation is based on the huge spot size of the GD-OES, the thicknesses are therefore stated as area averages in tens digit. The Fe, Mn, and Cu signals reach a plateau within the first second. After immersion in TSB for 24 h (Figure 2C,D), the oxide layer increases due to corrosion processes up to about 130 and 300 nm for FeMnC and FeMnCCu, respectively. Clearly, the addition of Cu leads to an increase in the formation of the degradation layer. Besides the main alloy elements and O, further elements were measured on the degraded FeMnC samples: N, H, and Ca. Interestingly, the C signal peak has a maximum at about 15 nm along with a peak of the Ca signal and small shoulders in the Fe and Mn signals. A very small peak of the K signal (curve not shown) is observed. Shortly thereafter, the O and H signals reach their peaks, and the N signal is more or less at a plateau at that point. Those signals decrease within the degradation layer until the bulk metal is reached. A slightly different elemental composition of the degradation layer was observed for FeMnCCu. However, again there is a C signal peak at about 30 nm accompanied by peaks of the Fe, Mn, Cu, Ca, and N signal. The maximum peak of the Cu signal is at a depth of about 230 nm (about 70 nm above the bulk material). The intensity of the Cu signal peak is about 1.3× higher than the Cu signal in the bulk, indicating an enrichment of Cu in the degradation layer. Another aspect is the ratio of the alloying elements in the bulk material compared to that of the degradation layer. Therefore, the ratio of the Mn-to-Fe signal intensities and the Cu-to-Fe signal intensity were plotted in Figure 2E. More Cu and less Mn are integrated in the degradation layer compared to Fe. This observation points to an enrichment of Cu and a depletion of Mn in the oxide layer.

**Figure 3:**
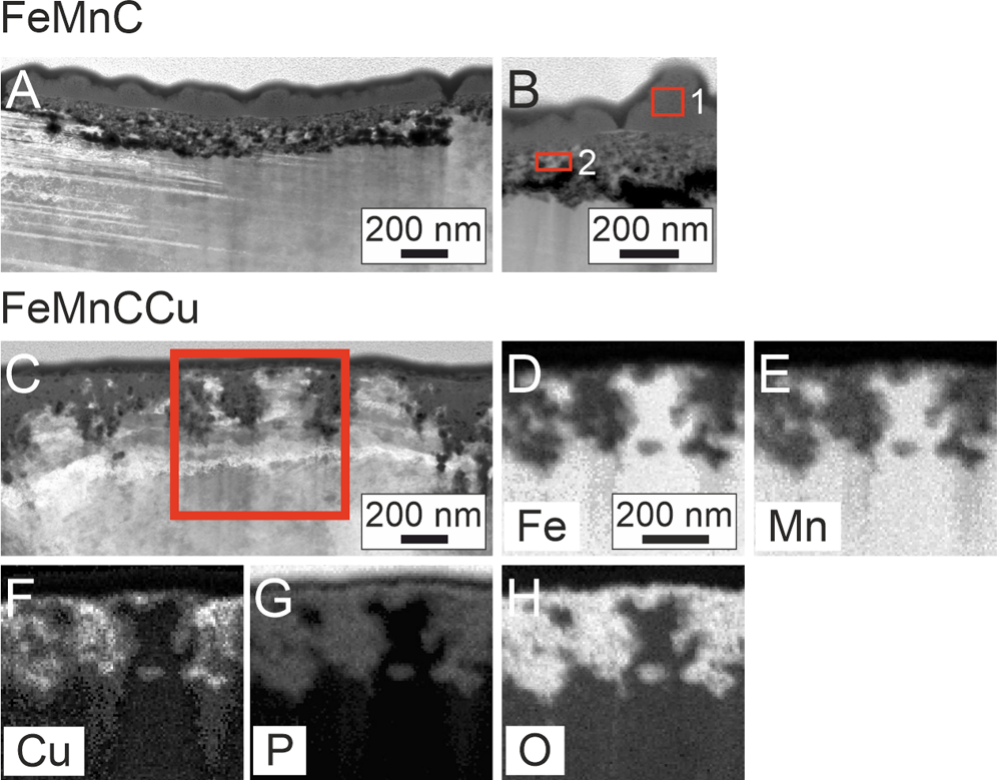
(A) TEM image showing the degradation layer of FeMnC after 24 h of immersion in TSB. (B) Higher magnification of the FeMnC degradation layer with red squares indicating the areas for elemental analysis (Table 3). (C) TEM image showing the degradation layer of FeMnCCu after 24 h incubation in TSB with the red square indicating the area of the corresponding EDX mappings of (D) Fe, (E) Mn, (F) Cu, (G) P, and (H) O. The brightness of the image reflects the increasing concentration of the respective element.

With transmission electron microscopy (TEM, Figure 3), the cross sections of the degradation layers were analyzed. It revealed a strong irregular degradation layer thickness of 155–470 and 45–750 nm for FeMnC and FeMnCCu, respectively. For FeMnC two distinct regions of the degradation layer could be identified (Figure 3B and Table 3). Both consisted of the elements Fe, Mn, C, and O. In the outer degradation layer also, P and very low signals of K and Ca were detected. This layer seems to be deposited onto the inner degradation layer with a sharp interface and an abrupt change from one degradation layer to the other. The inner degradation layer appears to start at the former bulk-to-atmosphere boundary, moving into the bulk material as degradation proceeds. However, the degradation layer of FeMnCCu had a quite different appearance without such clear separation of two different regions within the degradation layer. It shows a more inhomogeneous degradation. Regions of high O and P seem to grow into the substrate as corrosion proceeds. The Cu diffused into regions of high corrosion activity forming among other Cu-enriched phosphates and fingerlike FeMn-rich regions within the degradation layer.

**Table 3:** Quantified TEM–EDX Measurements of Selected Element Concentrations in the Degradation Layer of FeMnC (Corresponding to Areas Indicated in Figure 3B) and FeMnCCu (Figure 3C)

degradation layer	Fe/wt %	Mn/wt %	Cu/wt %	O/wt %	P/wt %	K/wt %	Ca/wt %
FeMnC (1) outer layer	16.6	4.1		56.6	8.7	0.5	0.8
FeMnC (2) inner layer	32.8	10.3		20.9	0.1		
FeMnCCu	17.3	8.5	8.6	44.4	3.4		

**Figure 4:**
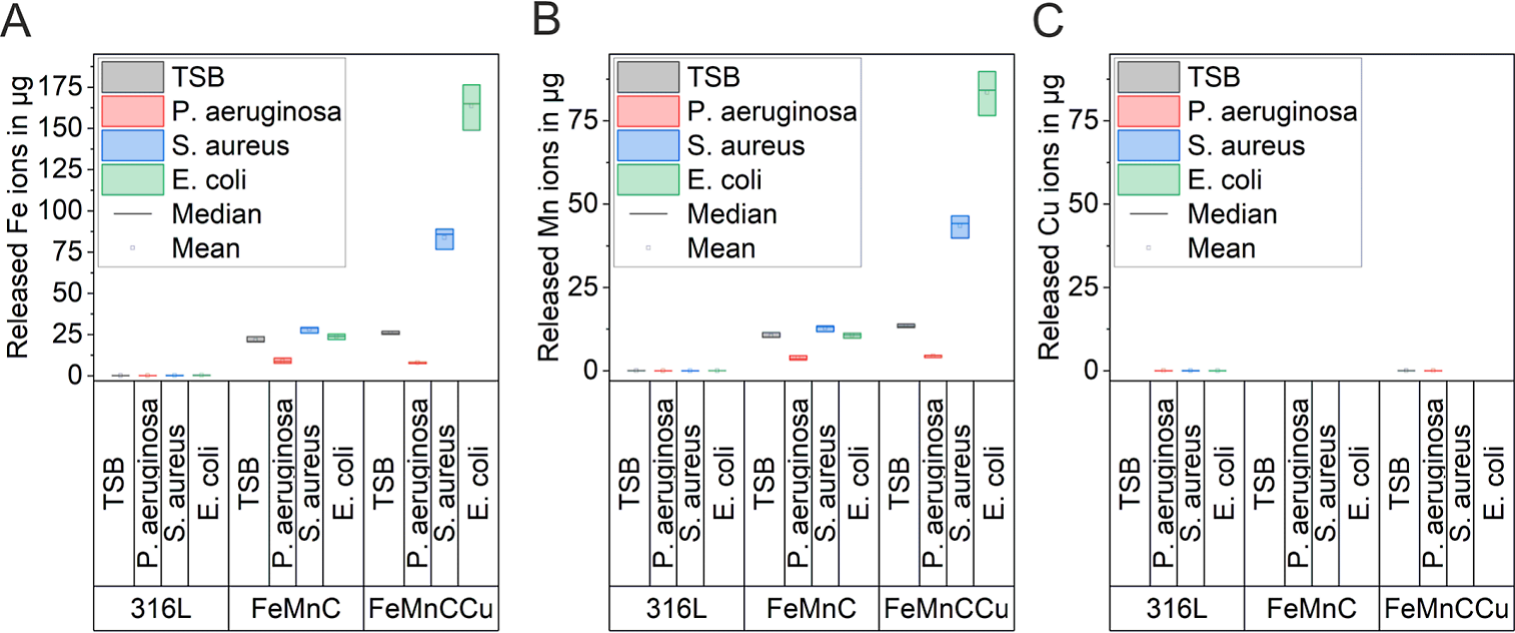
Concentration of released (A) Fe, (B) Mn, and (C) Cu ions of Cu-containing 316L, FeMnC, and FeMnCCu after 72 h in a TSB medium without (gray) and with cultivation of *P. aeruginosa* (red), *S. aureus* (blue), and *E. coli* (green). Please note the different scales on the *y* axis.

In Figure 4, the release of Fe, Mn, and Cu ions after 72 h of incubation in the TSB electrolyte was measured by ICP-OES. Ion release is shown in the presence of *P. aeruginosa*, *S. aureus,* and *E. coli*, and also without the cultivation of bacteria (referred to as TSB). For comparison reasons, the possible release of Cu ions was investigated in TSB electrolytes of all three investigated materials. As FeMnC was not alloyed with Cu, no Cu ions were detected in the solution. For both FeMnCCu with 3.5 wt % Cu and 316L with 0.4 wt %, the detected Cu release is either very low, about 0.03 μg, or below the detection limit of the applied ICP-OES. Furthermore, the Fe and Mn ion release of the 316L reference is very low near the detection limit of ICP-OES. This low ion release is expected for 316L as the material exhibits a very low corrosion density in the PDP measurements due to spontaneous passivation. However, for FeMnCCu, a measurable Cu ion release was expected due to the active dissolution behavior of this alloy, which was revealed in PDP measurements.

In the TSB electrolyte without cultivation of bacteria, a significantly higher release with *p* < 0.05 of both Fe and Mn ions for both biodegradable FeMnC-based alloys was observed compared to reference 316L stainless steel. The release of Fe ions is in both FeMnC-based alloys higher than the release of Mn ions due to the higher Fe concentration in the alloys. Due to alloying with Cu the release of both Fe and Mn ions is significantly (*p* < 0.05) higher than in FeMnC. Additionally, an influence of the presence of bacteria on ion release was found. For the FeMnC, a slight decrease of the Fe and Mn ion release was seen in the presence of *P. aeruginosa*, an increase in the presence of *S. aureus* and almost no influence was seen in the presence of *E. coli* compared to TSB. However, the Cu-modified alloy shows a quite different behavior. The Fe and Mn ion release is significantly increased in the presence of *S. aureus* and *E. coli*. The presence of *P. aeruginosa* leads to a similar decrease of Fe and Mn ion concentration, as seen for the FeMnC. The Fe and Mn ion release of the 316L reference is not influenced by the presence of bacteria.

#### 3.2.2 Quantification of Attached Viable Cells on FeMnC-Based Alloys

**Figure 5:**
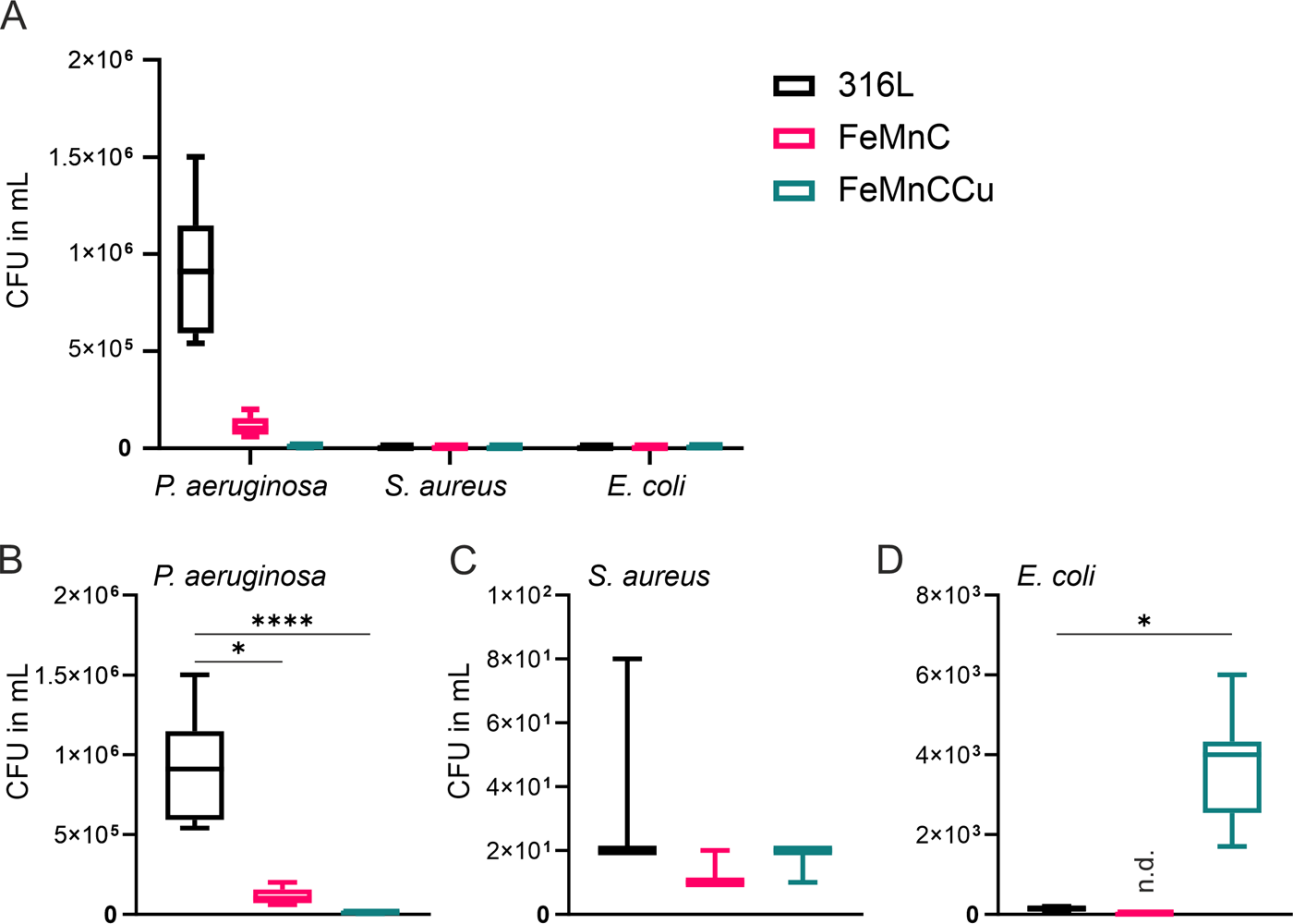
Quantification of viable cell attachment of *P. aeruginosa*, *S. aureus,* and *E. coli* cultivated in TSB for 72 h on 316L, FeMnC, and FeMnCCu by colony forming unit (CFU) assays. (A) All bacteria were plotted together, revealing high CFU/mL values for *P. aeruginosa*. For a better understanding of the distribution, (B) *P. aeruginosa*, (C) *S. aureus,* and (D) *E. coli* were plotted individually with different *Y*-axis scales. Means and standard errors of the mean are plotted and are statistically analyzed by the Kruskal–Wallis test [*p* < 0.05 (*), *p* < 0.0001 (****)].

In order to gain insights into bacterial attachment up to biofilm formation on the two FeMnC-based alloys, samples were incubated for 72 h in TSB with the presence of *P. aeruginosa*, *S. aureus,* or *E. coli*. For a quantification of viable cells attached to the surfaces, the CFU assay was performed (Figure 5). The corrosion-resistant 316L stainless steel was used as the reference material. In Figure 5A, the results for all of the tested bacteria are depicted. The *P. aeruginosa* wild strain reveals the highest values of viable colonies of all tested bacteria with about 9 × 10^5^ CFU/mL (Figure 5B) on the reference 316L which is around six decades higher compared to *S. aureus* and *E. coli*. In order to observe the attachment distribution of the tested bacteria on the different alloys, they were additionally plotted individually (Figure 5B–D). A significant reduction in the number of viable colonies is observed for the FeMnC-based alloys. About 1 × 10^5^ CFU/mL were detected on FeMnC. Especially for the FeMnCCu, a growth-inhibiting effect is evident for *P. aeruginosa* (about 8 × 10^4^ CFU/mL).

For *S. aureus*, a similar tendency between the reference material and the FeMnC-based alloys is observed. Most viable colonies grew on the 316L steel with about 40 CFU/mL. The CFU values observed for the two FeMnC-based alloys are approximately 50% lower. Nonetheless, the number of viable colonies for *S. aureus* is very low compared to *P. aeruginosa* and *E. coli*.

Interestingly, this growth inhibitory effect of the two tested FeMnC-based alloys was not observed for *E. coli*. The most viable *E. coli* colonies were found on the FeMnCCu, with about 3 × 10^3^ CFU/mL. In the reference material, only 135 CFU/mL could be detected. No viable colonies were counted on FeMnC.

#### 3.2.3 Structural Observation of Bacterial Attachment and Biofilm Production of FeMnC-Based Alloys

**Figure 6:**
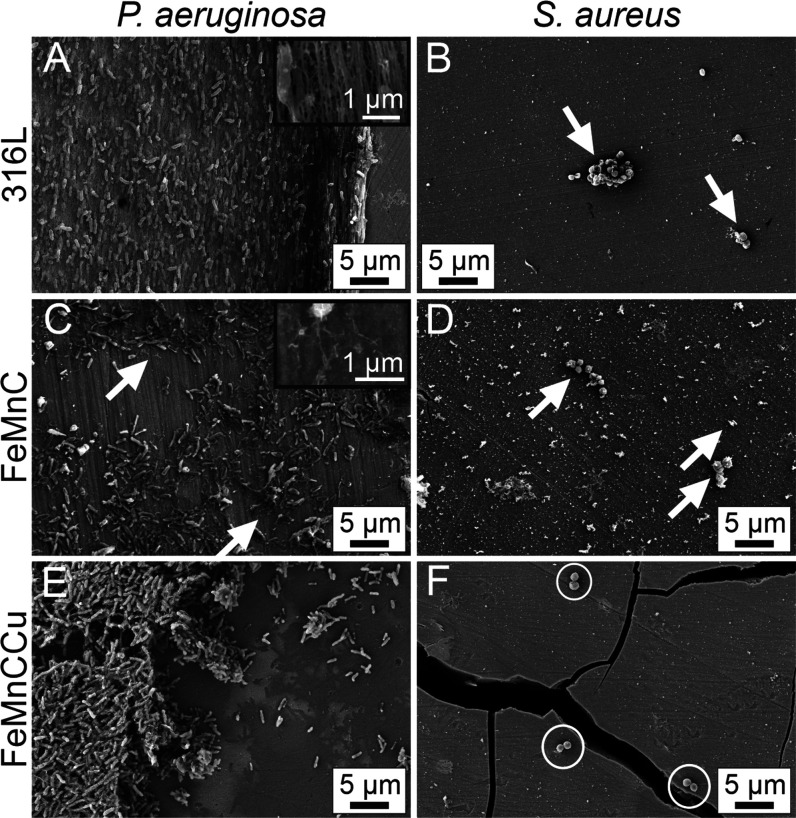
Structural observation of bacterial attachment and biofilm formation on 316L, FeMnC, and FeMnCCu. SEM images of (A,C,E) *P. aeruginosa* and (B,D,F) *S. aureus* performed after cultivation for 72 h on 316L, FeMnC, and FeMnCCu. (A) Inset of biofilm. (C) White arrows indicate residues of biofilm (inset). (B,D) White arrows indicate clusters of cells. (F) White circles indicate paired bacteria cells.

**Figure 7:**
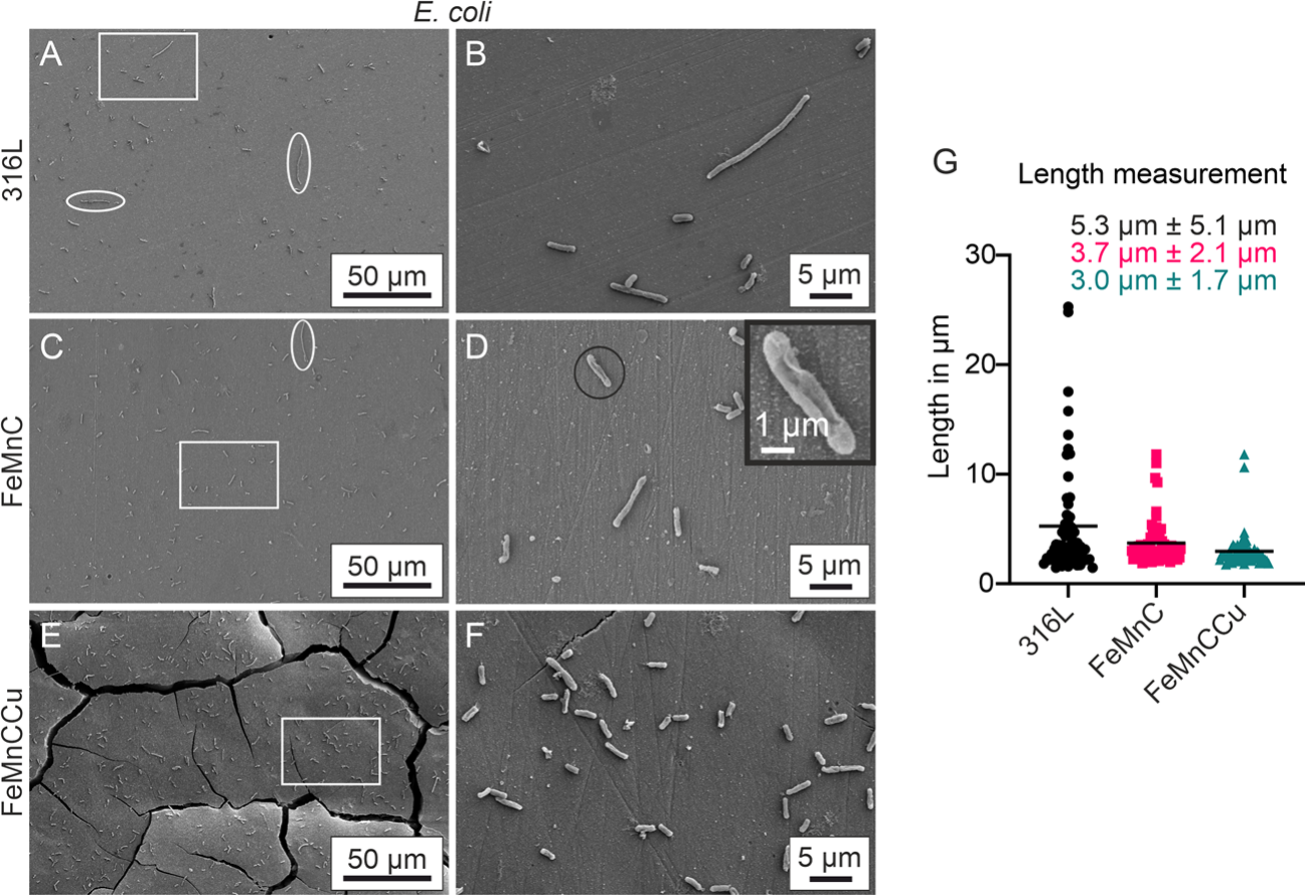
Structural observation of *E. coli* attached to (A,B) 316L, (C,D) FeMnC, and (E,F) FeMnCCu. The first column (A,C,E) shows overview SEM images to observe the bacterial distribution; white ellipses highlight long bacterial cells, indicating cell stress. White rectangles represent the areas which are shown at a higher magnification in the second column (B,D,F). (D) Black circle and inset show a shrunken, collapsed cell. (G) Scatter plot of *E. coli* cell length attached to the three different alloys (*n* = 60 for each alloy). Black lines represent the mean value. Written values constitute mean values with the standard deviation.

For a further insight into bacterial attachment and biofilm formation of *P. aeruginosa*, *S. aureus,* and *E. coli*, SEM was performed (Figures 6 and 7). For *P. aeruginosa*, a bacterial attachment was visible on all of the tested materials. However, a uniform surface-covering biofilm was only observed on the 316L steel (Figure 6A). Many cells are oriented parallel to the outer film boundary on the right sight of the image. The cells on the top of the film are randomly oriented. Beside the multilayered, three-dimensional film structure, even a slime layer consisting of polymeric substances was already formed as a holey grid with irregularly constituted meshes, which is visualized with a lower contrast as the cells themselves (Figure 6A inset). The *P. aeruginosa* cells attached to FeMnC appear rather individually (Figure 6C) and form only a loosely bound, rarely multilayered, 2D network. Residues of the network structures or rather the polymeric substances of the extracellular matrix were observed (Figure 6C white arrows and inset). On the FeMnCCu, the cells are organized in a highly porous, slightly connected three-dimensional network (Figure 6E). A textured growth or even an orientation of cells along the growth boundary is absent, and the growth is therefore substantially different compared to the 316L sample. The dense holey grid is mainly reduced to fibers which connect some of the cells with each other and again differs from the observations made on the 316L.

For Gram-positive *S. aureus*, no surface covering growth was monitored on any of the tested samples. Only some aggregates with a countable number of cells were formed on the 316L steel and the FeMnC (Figure 6B,D). No biofilm formation was found, which is clearly different from *P. aeruginosa* growth behavior on the 316L steel and the FeMnC. However, clusters of various *S. aureus* were identified (Figure 6B,D white arrows). It should be noted, that the bacterial cells keep their typical spherical shape. The suppressed growth of *S. aureus* turns out even more drastically on the FeMnCCu sample. Here, only isolated, often paired, bacteria cells were observed on the surface (Figure 6F white circles). No tendency to form a multilayer film or any kind of network is noticed. These few visible cells on the surface are consistent with the results obtained from the CFU assay.

The structural observation of the attachment of *E. coli* revealed no surface covering growth on the different alloys (Figure 7A–F). From SEM images, a similar amount of adherent cells is visible on FeMnC and FeMnCCu (Figure 7C,E). However, compared to FeMnCCu, larger areas of the FeMnC samples were not covered with cells. This is consistent with the CFU results (Figure 5D). Interestingly, the cell length increased on both 316L and FeMnC, indicating cell stress and upregulation of SOS functions, a cell response to DNA damage (Figure 7A,C white ellipses). To verify this assumption, cell lengths were evaluated and plotted (Figure 7G). The longest cells were found on the 316L steel (mean cell length 5.3 ± 5.1 μm), where they reached lengths of up to 25 μm. This suggests that stress-induced reactions do not arise on the two FeMnC-based alloys, and bacteria cell death rarely occurs. However, the trend for shorter cells points to FeMnCCu, which is supported by the CFU results revealing the most viable colonies on FeMnCCu.

From these SEM investigations, it is also clear to see that due to Cu alloying the degradation layer increased in volume as the surface of FeMnCCu (Figures 6F and 7E,F) displayed huge cracking compared to FeMnC (Figures 6C,D and 7C,D).

## 4 Discussion

One approach to decrease implant-related, pre- and postoperative infections in current research is alloying with Cu. In this study, 3.5 wt % Cu was added to the biodegradable Fe69Mn30C1 alloy to gain improved antibacterial properties besides maintaining a single-phase austenitic microstructure, which is desired for a homogeneous, controllable degradation and related mechanical integrity. Furthermore, this single-phase austenitic matrix is desirable for magnetic resonance imaging (MRI) compatibility as austenite might be antiferromagnetic^35^ depending on their Néel temperature.

The microstructure of the Cu-containing alloy was single-phase and austenitic (Figure 1A), which was also seen for FeMnC-based alloys with 0.8–1.5 wt % Cu after heat treatment.^37,38^ However, the LeBail fitting revealed a second austenitic phase with a slightly smaller lattice parameter in one of the FeMnC samples. This can originate from segregation which were revealed in SEM–EDX analyses (Figure 1D–L) with Mn enriched in interdendritic regions and a Fe-enriched matrix in dendritic regions. A similar effect of Fe and Mn segregation in FeMnC on the formation of two austenitic phases with slightly different lattice parameters was also seen in another study.^49^

### 4.1 Influence of Cu Addition on Microstructure and Corrosion

For investigating the initial corrosion behavior of the biodegradable FeMnC-based alloys in TSB electrolyte compared to the nondegradable, clinically applied 316L stainless steel, PDP measurements were performed (Figure 1C). Regarding the corrosion potentials, as Cu has a higher corrosion potential than Fe, the Cu alloying increases the corrosion potential of FeMnC. A similar effect was also seen for other Cu-containing FeMnC-based alloys in Na_2_SO_4_ solution,^50^ pure Fe,^34,51^ or FeMn^36^ in HBSS. PDP curves of the two biodegradable FeMnC-based alloys show almost similar electrochemical behavior. The possible local microgalvanic element formation between Cu-enriched regions and FeMn-rich regions did not have a clear global effect on the corrosion current density in the PDP measurements. Compared to the 316L steel, biodegradable alloys in general exhibit about one to three orders of magnitude higher current densities.^41^ The change in the slope of the anodic curve branch can be assigned to a change in the complex reaction mechanism. This was described for pure Fe in chloride-containing solutions where the dissolution of pure Fe at low polarization is controlled by chloride and hydroxide ions.^52^ With the change in slope at higher potential the dissolution rate is more than executed by only hydroxide ions.

**Figure 8:**
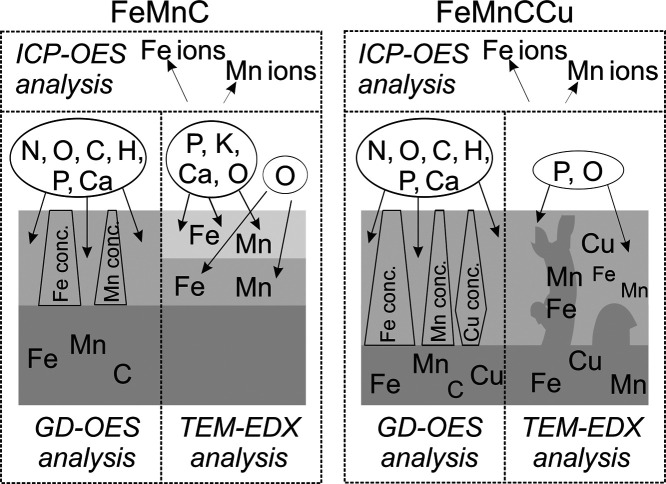
Scheme summarizing the results of the ICP-OES, GD-OES, and TEM–EDX analyses of FeMnC and FeMnCCu after 1 day (or 3 days for ICP-OES analyses) of incubation in TSB electrolyte.

After static immersion in the TSB electrolyte at neutral pH, the degradation layer was analyzed by GD-OES and TEM (Figure 8). As it was shown by previous studies, the corrosion of Fe-based alloys in complex simulated body fluids slows down significantly in early stage due to, e.g., phosphate/carbonate deposition, oxide formation or adsorption of proteins.^53,54^ This was also seen for both FeMnC-based alloys in the TSB electrolyte as both oxide layers increased rapidly after 1 day of immersion. In contrast to the PDP measurements, GD-OES and TEM analysis revealed an influence of the Cu addition (Figures 2 and 3). The alloying resulted in a thicker and quite porous degradation layer with lots of cracks on the surface (Figures 6F and 7E) which was also observed in HBSS.^35^ A global thickness of 300 nm compared to 130 nm for FeMnC was measured with GD-OES. However, TEM analysis of the cross sections of both degradation layers could not clearly confirm the increase of the layer thickness due to Cu (Figure 3) as the layer thickness of both alloys was very heterogeneous. These differences are caused by the difference in the analyzed area. For GD-OES, an area of 2.5 mm in diameter was investigated, whereas the imaged region in TEM was only a few μm^2^. Yet, TEM (Figure 3C) also revealed a more porous degradation layer due to Cu alloying. Furthermore, the structure of the degradation layers differed between both alloys. The FeMnC seems to have two distinct regions within the degradation layer as visualized by the TEM images (Figure 3A,B). The outer phosphate-rich degradation layer seems to grow in cauliflower-shape onto the inner degradation layer which mainly consists of oxides. This was not clearly visible within the degradation layer of the FeMnCCu where branching columnar structures were observable instead.

Furthermore, the Mn concentration is depleted in the oxide layer of both alloys (Figure 2E) as seen before for another FeMnC-based alloy.^55^ An enrichment of Cu in the degradation layer was measured with a concentration about 35% higher than that in the bulk sample regarding GD-OES data, which was confirmed by EDX measurements from the TEM (Figure 3). An increased local corrosion due to microgalvanic coupling of FeMn-rich to Cu-enriched regions might be an explanation. Such an enrichment of Cu in the oxide layer is known for Cu-containing FeMnC-based steels.^50^ Although an effect of the Cu addition on the corrosion rate could not be specified in that study.^50^ The authors also found a large heterogeneity and an increase in the thickness of the oxide layer due to Cu as an alloy ingredient. However, for a clear validation of the local effect of Cu, time-dependent degradation experiments would be necessary.

Additionally, ICP-OES analysis could not measure Cu ions in the electrolyte but rather an increased Mn and Fe ion concentration due to the Cu alloying. The FeMn-based alloy, the electrolyte, and surface area-to-electrolyte volume ratio highly influence the measurable Cu ion release concentration. In this study, a surface area-to-electrolyte volume ratio of about 0.007 cm^2^ per 1 mL electrolyte was chosen to reach the conditions used for previous cytocompatibility cell studies.^49,55^ Also, Mandal et al.^35^ could not detect a release of the Cu ion in HBSS from FeMn-based alloys using a higher ratio of 0.05 cm^2^ sample surface per mL electrolyte. However, Guo et al.^34^ found an increase of Cu ion concentration for biodegradable Fe-based alloys in HBSS with an increasing Cu (1.5–7.8 wt %) alloy concentration using a very high ratio of 3 cm^2^ per mL electrolyte. Another aspect is the Mn-to-Fe concentration ratio, which was slightly higher in the electrolyte than in the bulk. However, this corresponds to GD-OES measurements indicating a Mn depletion of the oxide layer, which was seen also for another FeMnC-based alloys.^55^

Overall, both metal ion release and degradation layer thickness indicate a higher corrosion rate of FeMnCCu due to local microgalvanic corrosion between the FeMn-matrix and Cu-rich regions in the degradation layer.

### 4.2 Antibacterial Effect of Biodegradable FeMnC-Based Alloy

In this study, the bacterial attachment and biofilm formation of three different bacteria strains on biodegradable FeMnC-based alloys was evaluated for the first time in comparison with corrosion-resistant 316L stainless steel. Studies investigating the antibacterial effect of Cu or Ag in Fe-based alloys, considered only one bacteria strain for their testing, either *E. coli*,^33−36^*S. aureus,*^37,56^ or both strains^57^ as well as various staphylococci strains.^58^ Therefore, FeMnC-based alloys were evaluated for the first time regarding their antibacterial behavior against *P. aeruginosa* in this study. Additionally, clinically applied AISI 316L stainless steel was used as reference material for all three bacterial strains in comparison to the FeMnC-based alloys, whereas other studies analyzing Cu-containing Fe-based alloys utilized only either pure Fe,^34^ FeMn,^36^ a positive control,^35^ or no control.^33^ Furthermore, those antibacterial tests were conducted with extracts of the respective alloys, whereas in this study, the bacterial cells were directly seeded onto the appropriate sample surface.

A significant reduction of the CFU was observed for the strong biofilm producer *P. aeruginosa* on FeMnC compared to 316L. For *S. aureus* and *E. coli*, the CFU number also decreased after 72 h of incubation but only slightly. However, no surface covering growth could be detected on any of the tested materials for *S. aureus* and *E. coli*. We conclude that FeMnC-based alloys have an inherent antibacterial effect. One antimicrobial component used here is iron.

Interestingly, it is demonstrated in the study of Xia et al.^17^ that the ion release of Fe-containing clays was also influenced by the bacterial growth. Compared with the used *E. coli* strain, the concentration of Fe and Mn ions in the presence of *P. aeruginosa* was significantly decreased. This behavior can be explained by the ion sorption capacity of the bacterial biofilm.^59^ Within this biofilm, cells secrete extracellular polymeric substances (EPS), which act as a protective layer and a diffusional barrier. The complex heterogeneous composition of the EPS matrix allows binding of both nutrients and external stressors and additionally influences the diffusion rate. The sorption system within the EPS matrix is able to accumulate potential toxic metal ions like Fe or Cu ions^59^ and in our study possibly also Mn ions. The present study verifies that *S. aureus* increases the Fe and Mn ion release and the dissolution of both ions in the medium. During this bacterial leaching, the reduction of Fe^3+^ to Fe^2+^ by *S. aureus* produces additional protons.^60^ Various bacterial species are known for their ability to leach FeMn ores or recycle FeMn waste.^61^ In the present study, this observation is extended to *S. aureus* and FeMn-based alloys. However, the increased ion release could also be a result of different pH values across the biofilms as the physical boundary of EPS can foster chemical gradients of, e.g., oxygen concentration or pH value.^62^

### 4.3 Effect of Cu Addition on Material–Bacteria Interaction

FeMnC has an inherent antibacterial effect on *S. aureus* and *P. aeruginosa*. This mostly relies on the formation of ROS.^17^ For the biodegradable FeMnC-based alloys, an antibacterial effect against *P. aeruginosa* was observed when the cells were in contact with the material surface. The addition of Cu to FeMnC resulted in a further decrease of the CFU number of *P. aeruginosa*. This observation is a quite prominent antibacterial effect for wild-type bacteria, like the *P. aeruginosa* strain used in this study, as these wild strains are more resistant than the equivalent from the strain collection.^63^ Yet, the Cu ion concentrations were below the detection limit in the various electrolytes; therefore, only very few Cu ions would be in the solution. These concentrations might be too low for a suitable antibacterial effect of Cu ions.^22^ Therefore, either the contact killing effect of Cu or the Fe and Mn ions absorbed in the biofilm and the corresponding ROS formation could be responsible for the decreasing CFU numbers. As ROS can catalyze the reduction of metal ions, such redox reactions can disaggregate bacterial cell membranes, followed by the oxidation of their nucleus, the release of their cytoplasm and finally the death of the bacterium.^22^ For deeper understanding, a more complex time-dependent analysis of bacterial growth in contact with the reaction products has to be executed. While ion leaching is much higher in alloys with Cu, for unknown reasons, FeMnCCu alloys allow a much higher CFU for *E. coli* and a cell shape which is in the physiological reach of 1–2 μm.^64^ Other studies which used extracts of FeCu-based alloys to investigate the bacterial behavior, revealed the generally expected increasing antibacterial effect against *E. coli* due to increasing Cu concentration in the alloy.^34,35^ However, it is not clear whether that effect is due to increased Fe or Cu ion release.

In summary, this present study proves an inherent antibacterial effect against *P. aeruginosa*, *S. aureus,* and *E. coli* of the here tested biodegradable FeMnC for the first time. *P. aeruginosa* is within the group named ESKAPE bacteria, which are the six mostly appearing antibacterial-resistant bacteria and is also associated with nosocomial device related infections such as ventilator associated pneumonia or urinary tract infections.^65−67^ Using the Cu-alloyed FeMnCCu, *P. aeruginosa* were killed efficiently. This result opens new avenues for the development of stents, where *P. aeruginosa* is a major cause of implant-related infections.

## 5 Conclusions

This study displays the influence of the alloy composition on the initial corrosion behavior, as well as on the microbial corrosion and related antibacterial behavior of biodegradable FeMnC(Cu) alloys.

Both biodegradable alloys display a single-phase, dendritic microstructure in the as-cast state. However, Cu has a significant influence on the degradation behavior as shown by immersion studies, which is quite promising. After 3 days of immersion in TSB electrolyte, subsequent ICP-OES studies revealed a significant increase of Fe and Mn ion release due to Cu alloying. Furthermore, GD-OES and TEM–EDX analyses after 1 day of immersion in TSB revealed an influence of Cu alloying on the degradation layer, which is more heterogeneous and porous with a globally increased thickness. Short-term electrochemical analysis (degradation within hours) could not confirm the effect of Cu alloying. To get more insights into the corrosion mechanisms, time-dependent corrosion studies have to follow to investigate the influence of Cu at different stages of corrosion and to get more insights into the corrosion mechanisms. Furthermore, perfusion experiments have to follow for better mapping of *in vivo* corrosion.

To the best of our knowledge, this preliminary study is the first of its kind comparing the antibacterial effect of biodegradable FeMnC(Cu) and clinically applied corrosion-resistant 316L stainless steel. An inherent antibacterial effect of FeMnC on *S. aureus*, *P. aeruginosa* and *E. coli* was revealed, most probably relying on the formation of ROS during corrosion which is quite promising for later application as a temporary implant material. Further studies with other bacterial strains should follow to verify these results.

Cu addition was expected to further enhance the antibacterial effect, as Cu is well-known for its antibacterial effect in various alloy systems. However, only for the Gram-negative, wild-type *P. aeruginosa*, a much more resistant strain than the equivalent from the strain collection, a further increase of the antibacterial behavior was observed. As *P. aeruginosa* is one of the main bacteria causing coronary stent infections, FeMnCCu has a high potential as temporary implant material for coronary stents. Furthermore, as the possible Cu ion release was below detection limit and a Cu enrichment in the degradation layer was seen with GD-OES and TEM-EDX, a contact killing effect of Cu could be assumed. However, *P. aeruginosa* led to a similar decreased Fe and Mn ion concentration in the electrolyte supernatant of both alloys. An increased absorbed Fe and Mn ion concentration in the biofilm on FeMnCCu is assumed. Therefore, these increased ion concentrations and corresponding increased ROS concentrations could be another explanation for the enhanced antibacterial effect of FeMnCCu against *P. aeruginosa*. More in-depth analyses have to follow to investigate the responsible mechanisms. For still unexplained reasons, *E. coli*—the second Gram-negative bacteria strain in this study—displayed a different behavior on FeMnCCu: a strong proliferation. Furthermore, *E. coli* triggered corrosion quite differently. While the ion concentration in the supernatant of FeMnC was not significantly influenced by *E. coli*, the Fe and Mn ion concentration in the supernatant of FeMnCCu was significantly higher compared to that in the supernatant without bacteria. This increased proliferation of *E. coli* with increased Fe and Mn ion release due to Cu alloying leaves the scope for following studies on the mechanisms of Cu ions on bacterial behavior. In further investigations, both FeMnC-based alloys should be also tested regarding cytocompatibility.

## Abbreviations

BSE: back scatter electron imaging
CFU: colony-forming unit
EDX: energy-dispersive X-ray analysis
FeMnC: Fe69Mn30C1
FeMnCCu: Fe65.5Mn30Cu3.5C1
FIB: focused ion beam microscope
GD-OES: glow discharge optical emission spectrometry
HBSS: Hank’s balanced salt solution
ICP-OES: inductively coupled plasma optical emission spectroscopy
MRI: magnetic resonance imaging
OCP: open circuit potential
PDP: potentiodynamic polarization
ROS: reactive oxygen species
SBF: simulated body fluid
SEM: scanning electron microscopy
TEM: transmission electron microscopy
TSB: tryptic soy broth with additional 1% glucose
XRD: X-ray diffraction analysis
